# Is there a ubiquitous association between sleep disorder and frailty? findings from LASI (2017–18)

**DOI:** 10.1186/s12877-023-04148-x

**Published:** 2023-07-12

**Authors:** Aditi, S. K. Singh, Ajit K. Jaiswal, Madhur Verma

**Affiliations:** 1 Department of Survey Research and Data Analytics, International Institute for Population Sciences, Mumbai, India; 2 Department of Fertility Studies, International Institute for Population Sciences, Mumbai, India; 3 Assistant Professor of Community/Family Medicine, All India Institute of Medical Sciences Bathinda (Punjab), Punjab, India

**Keywords:** Sleep disorder, Frailty, Older adults, LASI, India

## Abstract

**Background:**

Relatively little is known about how sleep disorders affect frailty of seniors. The study uses Fried's frailty index, to investigate the relationship between sleep disorder and frailty among older Indian adults.

**Methods:**

The study analysed Longitudinal Ageing Study in India (2017–18) data which uses a multistage stratified area probability cluster sampling design. The association between frailty was studied for which the total sample size was 31,902. The principal dependent variable was frailty. Descriptive statistics and cross-tabulation were presented in the study. A binary logistic regression analysis was used to fulfil the study objectives to find the possible association.

**Results:**

The prevalence of frailty in India was 21.3 percent. Older adults with sleep disorder had 66 percent higher likelihood to be frail than their counterparts. The benefits of physical activity in containing frailty is huge, the association were quite high. Poor Self-rated health was significantly associated with higher frailty (OR = 1.73; CI = 1.47–2.04).

**Conclusions:**

Frailty is an enormously growing public health issue and has bi-directional relation with sleep disorders. The study has clinical relevance since sleep complaints offer a means for identifying those who are vulnerable to frailty and through appropriate intervention, the causes of sleep disorder would help to delay and in some cases reverse frailty.

**Supplementary Information:**

The online version contains supplementary material available at 10.1186/s12877-023-04148-x.

## Background

The world’s older adult population is increasing rapidly; by 2050, the 60-plus population is expected to reach 2.1 billion [1]. As per Census 2011, around 8 percent of the Indian population was old, and this is expected to be double by 2050, which is 19.6 percent. By the end of the century, they will be nearly 34 percent of the total population [2]. Frailty, defined as the presence of multisystem impairment and expanding vulnerability, is accompanied by ageing and defined features like impaired gait speed, lower physical activity, tiredness/ exhaustion, diminished grip strength, and eventual weight loss. It is a common age-related, multidimensional condition that often leads to adverse outcomes, including mortality [3, 4]. The symptoms of frailty clearly signal a progressive decline in function as a person ages. It has been widely recognised that physical activity can have an impact on different components of the frailty [5]. It has a prevalence of about 10 percent in Indian community-dwelling elderly population [3]. Among the older adults aged more than 85 years, the prevalence is around 30 percent [2]. Sleep is one of the most important lifestyle processes of a human being, its duration and quality are crucial determinants of one’s health; this is even more critical in the elderly since ageing is accompanied by a substantial shift in sleep architecture [6]. As individuals age, they face numerous health challenges, and sleep disturbance supersedes all of those [7, 8]. In the broader sense, this manifests as a sleep disorder which is an alteration in the sleep routine that interferes with a person’s daily physical, mental, and emotional functioning [9]. Though it is highly prevalent and has detrimental effects on health; however, it remains poorly identified. The actual prevalence of sleep disorders remains unknown since various underlying diseases lead to changes in sleep patterns, yet reports of disrupted sleep are common in older adults with a frequency of more than 60 percent [10, 11].

The associations between sleep disturbances and frailty status in older people are ambiguous. Older adults having healthy relationships in the family and in society have a better quality of sleep; this also holds for keeping the prevalence of frailty low [12, 13]. Further, sleep disorders lead to functional decline, decreased muscle strength, decreased growth hormone levels, low physical activity, anxiety, and even falls. Since these are also the pathogenesis of frailty, it was postulated that sleep disturbances could be associated with frailty [14, 15]. Previous studies have also discussed the possible relationship of sleep disturbance being a risk of frailty [2, 9, 16–18]. Studies have shown that older adults categorised as frail were more likely to be female, people having lower education level, lower income group, having trouble with ADL and IADL, low physical activity, and have various comorbidities [8, 10, 19–22]. Frailty and sleep disorders are also significant precursors of functional deterioration leading to recurrent hospitalisation.

There is a dearth of literature from India about the profile of sleep disorders and associated frailty. Sleep–wake disturbance and even frailty, if intervened properly, are potentially remediable [23]. It is important to identify the risk factors of frailty and examine their association with sleep disorders, especially in India, where the number of older adults is increasing at an unprecedented rate. This would address preventing advanced frail conditions, such as disability and mortality and help in the management of specific underlying causes. With this, the study aimed to investigate the relationship between sleep disorder and frailty among older Indian adults. It is hypothesised for the study that there is a significant positive association between sleep disorder and frailty.

## Methods

### Data

This study uses data from the Longitudinal Ageing Study in India- wave 1 (LASI-2017–18). The nationally-representative sample in the survey included 72,250 individuals aged 45 and above and their spouses across all states and union territories of India except Sikkim. LASI adopted a multistage stratified area probability cluster sampling design to select the eventual units of observation. Households with at least one member 45 years and above were taken as the sample unit. All married and non-married men and women age 45 and above and their spouses in selected households were interviewed for the data. The data provides strong scientific evidence on demographics, household economic status, chronic health characteristics, symptom-based health conditions, biomarkers, work, and employment etc.

Detailed information is available on the LASI Wave-1 Report [24]. The sample size for the present study is 31,902 older adults, 14,652 older men and 15,899 older women aged 60 and above.

### Variable description

#### Outcome variable

Frailty among older adults was assessed using the Frailty Phenotype widely used in the many research on older adults [3]. The index is composed of five main components: self-reported exhaustion, weak grip strength, self-reported low physical activity, unintentional weight loss, and slow walking time.

The first component exhaustion was examined using the questions from the Center for Epidemiologic Studies Depression (CES-D) scale, from the LASI questionnaire: in the past week, how often do you feel “feel tired or low in energy’ and that, “everything you did was an effort,”. The respondent’s self-reported answer was “three or more days coded as 1” and “less than three days coded as 0”.

The second component of index was handgrip strength assessed using the measured readings of handheld Smedley’s Hand Dynamometer. The LASI data provides handgrip strength measured in kilograms. The outcome handgrip strength score (in kg) was calculated as the average score (in kg) of two successive trials in the dominant hand, and was adjusted for both the sexes and body mass index considering the difference that exists between various groups.

The third component of index- unintentional weight loss was composed using the proxy self-reported question: “Do you think that you have lost weight in the last 12 months because there was not enough food at your household?” with answers “Yes coded as 1” and “No coded as 0.”.

For the fourth component of physical activity, the study used the question: “How often do you take part in sports or vigorous activities, such as running or jogging, swimming, going to a health center or gym, cycling, or digging with a spade or shovel, heavy lifting, chopping, farm work, fast bicycling, cycling with loads: which was answered as every day, more than once a week, once a week, one to three times a month, or hardly ever or never” Finally, low physical activity was taken as: “One to three times a month or hardly ever or never = 1” and “once a week or more than once a week = 0” [25].

For the final and fifth component, the study used the average measured readings of respondent’s 4 m walk, and slowness was assessed by the time (in seconds) taken to cover the 4 m walk (also stratified by gender and height). The final Frailty Index score ranges between 0 and 5. Respondents in the study with a score of 0, were classified as “robust” 1–2 as “pre-frail” and those with a score of three or more were considered as “frail”. For the study, only the frail respondents were studied.

### Descriptive variables

#### Key independent variable

The variable of sleep disorder assessment was derived by combining three proximate questions, from the LASI questionnaire- 1 which was taken from the Jenkins Sleep Scale (JSS-4) [26]. How often do you have trouble falling asleep? 2. How often did you wake up too early in the morning and were not being able to fall asleep again? 3. How often did you wake up during the night and have trouble getting back to sleep? The response was reported as a. never, b. rarely (1–2 nights per week), c. occasionally (3–4 nights per week) and d. frequently (5 or more nights per week). Never/Rarely was recoded as 0 and “no,” and Occasionally/Frequently was recoded as 1 “yes”. If the respondent reported having any of the above problems, he/she was defined as suffering from a sleep disorder.

All the other independent variables are there in Supplementary Table 1.

**Table 1:** Socio-economic and demographic profile of the study population in India, LASI 2017–18

Background characteristics	Male	Female	Total
**Sleep disorder**			
No	68.1	59.7	63.7
Yes	31.9	40.3	36.3
**Individual factors**			
** Age**			
Young-old	57.8	59.1	58.5
Old-old	31.1	29.3	30.2
Oldest-old	11.0	11.5	11.3
** Education**			
Up to primary	47.8	60.7	52.1
Up to Secondary	40.4	33.8	38.2
Graduation & above	11.8	5.5	9.7
** Living arrangements**			
Alone	6.4	16.0	11.4
With spouse	54.4	28.2	40.6
With children	39.2	55.9	48.0
** Marital status**			
Currently married	81.1	44.1	61.6
Widowed	16.5	54.0	36.2
Others	2.4	1.9	2.2
** Working status**			
Yes	45.5	35.7	41.8
No	54.5	64.3	58.2
** Social engagement**			
1	36.8	26.0	31.1
2	26.8	32.2	29.7
3	36.4	41.8	39.3
**Behavioural factors**			
** Tobacco consumption**			
No	60.0	22.5	40.2
Yes	40.0	77.5	59.8
** Alcohol consumption**			
No	28.0	2.6	14.6
Yes	72.0	97.4	85.4
** Physical activity**			
Frequently	32.6	21.9	27.0
Rare/Never	67.4	78.1	73.0
**Health factors**			
** Body mass index**			
Underweight	28.2	25.2	26.7
Normal	41.3	35.5	38.3
Overweight	12.8	13.0	12.9
Obese	17.6	26.3	22.1
** Self-rated health**			
Good	33.9	27.8	30.7
Fair	43.9	46.2	45.1
Poor	22.2	26.0	24.2
** Difficulty in ADL**			
No	79.1	73.6	76.2
Yes	20.9	26.4	23.8
** Difficulty in IADL**			
No	34.4	26.6	30.3
Yes	65.6	73.4	69.7
** Morbidity status**			
0	49.7	44.4	46.9
1	28.1	30.1	29.2
2 +	22.2	25.4	23.9
**Household factors**			
** MPCE quintile**			
Poorest	20.8	22.5	21.7
Poorer	21.3	22.1	21.7
Middle	21.6	20.4	20.9
Richer	19.2	19.2	19.2
Richest	17.0	15.9	16.4
** Religion**			
Hindu	82.0	82.4	82.2
Muslim	11.7	10.9	11.3
Christian	2.6	3.1	2.9
Others	3.7	3.6	3.6
** Caste**			
Scheduled Caste	19.3	19.6	19.4
Scheduled Tribe	7.9	8.7	8.3
Other Backward Class	47.0	45.9	46.5
Others	25.8	25.8	25.8
** Place of residence**			
Rural	72.1	69.2	70.5
Urban	27.9	30.8	29.5
** Region**			
North	12.3	12.8	12.6
Central	22.5	19.6	20.9
East	24.6	22.8	23.6
Northeast	2.9	3.1	3.0
West	16.3	18.0	17.2
South	21.4	23.8	22.7
**Total**	**15,139**	**16,763**	**31,902**

*ADL* Activities of Daily Living, *IADL* Instrumental Activities of Daily Living, *MPCE* Per Capita Monthly Consumption Expenditure

#### Statistical analysis

Descriptive statistics and cross-tabulation were analysed for the study [27]. A binary logistic regression analysis [28] was done to assess the association between the dependent variable (frailty) and sleep disorder among older adults in India. To check for the multicolinarity problem in the data, Variance inflation factor (VIF) was used [29, 30]. For the comple analyses, STATA 16 was used. Map was made using GeoDa 1.18.

## Results

Table 1 represents the socio-economic and demographic profile of the study population. Out of total 31,902 older adults (15,139 males and 16,763 females), about 32 percent and 40 percent older males and females had sleep disorder respectively. Nearly, 48 percent and 61 percent of older males and females had no education or had primary education not completed respectively. About 16.5 percent and 53.5 percent of older men and women were widowed.

**Fig. 1:**
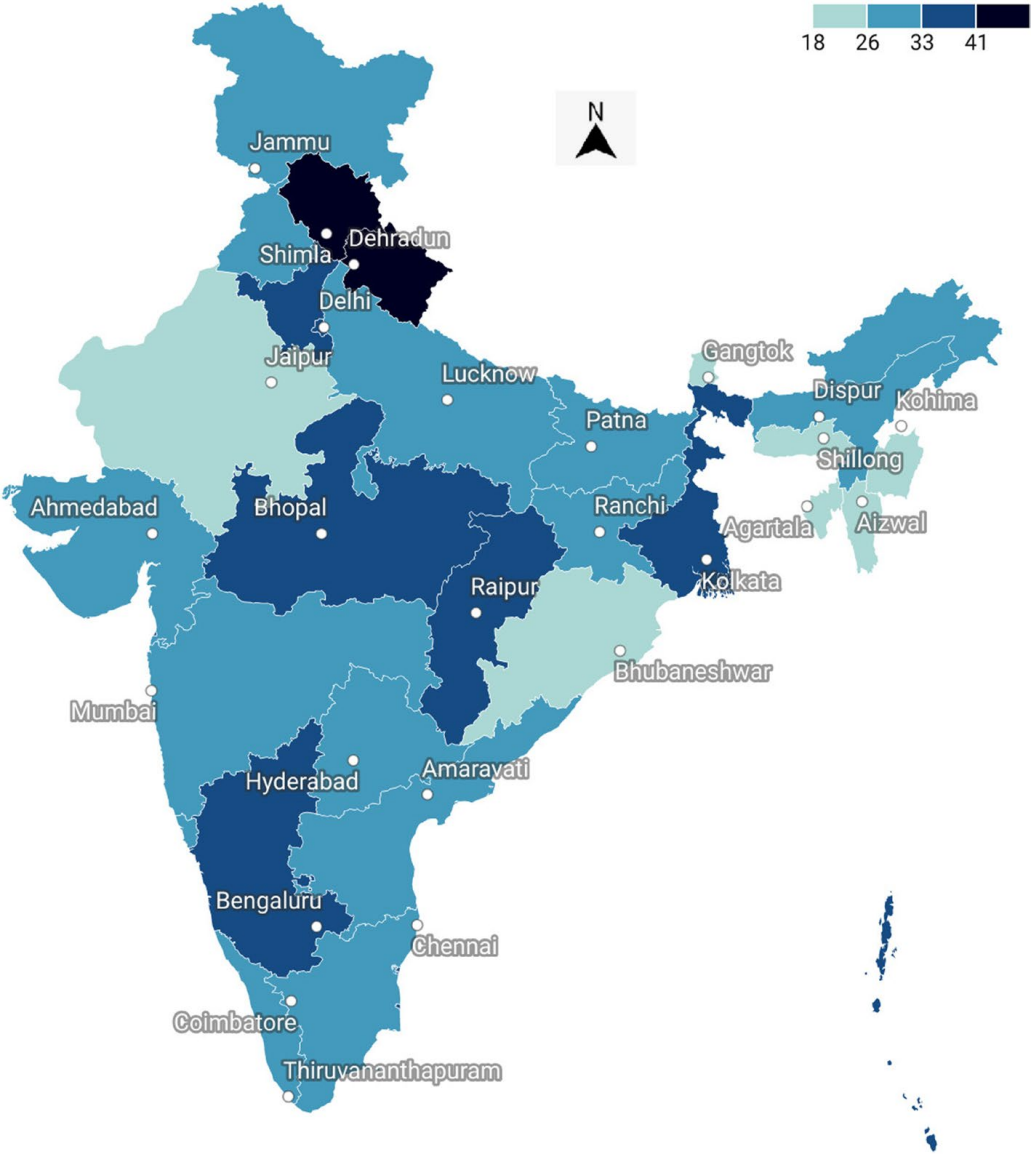
Prevalence of sleep disorders across various states of India findings from LASI (2017–18)

Figure 1 shows the prevalence of sleep disorders across various states of India. The Highest prevalence was found in the states of Himachal Pradesh (45 percent), Uttarakhand (38 percent), West Bengal and Madhya Pradesh (32 percent).

**Fig. 2:**
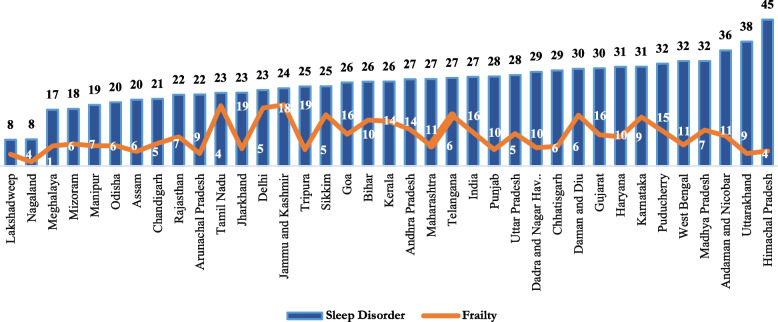
Pattern of sleep disorder and frailty across various states of India, LASI (2017–18)

Figure 2 is a representation of sleep disorder and frailty both for various states of the country. The lowest prevalence of sleep disorder was observed in the state of Nagaland (8 percent) which is still pretty higher.

Table 2 shows the percentage of older adults suffering from frailty in India in 2017–18 across the genders and as per different background characteristics. The results show the prevalence of frailty in the population to be 21.3 percent. The percentage of frailty increases with age, with the highest percentage being among the oldest-old (29.1 percent for males and 28.9 percent for females). Sleep disorders were found 30 percent and 27 percent amongst frail males and females respectively. Older adults in rural areas had a higher prevalence of frailty compared to those in urban areas, this was uniform across both the genders.

**Table 2:** Percentage of older adults suffering from frailty in India, LASI 2017–18

Background characteristics	Male	Female	Differences	*P*-value
	Percent	Percent	Percent	
**Sleep disorder**				
No	16.6	17.0	0.4	0.119
Yes	30.4	29.6	-0.8	0.096
**Individual factors**				
** Age**				
Young-old	17.0	18.0	1.0	0.000
Old-old	25.6	27.6	2.0	0.001
Oldest-old	29.1	28.9	-0.2	0.876
** Education**				
Upto primary	24.1	18.8	-5.3	0.463
Upto Secondary	16.4	13.2	-3.2	0.215
Graduation & above	11.1	16.4	5.2	0.316
** Living arrangements**				
Alone	26.6	28.9	2.4	0.159
With spouse	18.9	18.0	-0.9	0.617
With children	23.0	22.1	-0.9	0.075
** Marital status**				
Currently married	19.7	19.0	-0.7	0.714
Widowed	26.6	24.8	-1.8	0.598
Others	26.0	14.1	-11.9	0.011
** Working status**				
Yes	14.0	13.7	-0.3	0.874
No	26.6	27.7	1.1	0.023
** Social engagement**				
1	24	26.0	2.0	0.027
2	18.5	17.0	-1.5	0.011
3	20.5	21.4	0.8	0.323
**Behavioural factors**				
** Tobacco consumption**				
No	21.5	22.8	1.2	0.038
Yes	20.2	21.8	1.6	0.000
** Alcohol consumption**				
No	20.7	19.9	-0.8	0.227
Yes	21.1	22.1	1.0	0.003
** Physical activity**				
Frequently	2.5	3.1	0.7	0.062
Rare/Never	30.0	27.3	-2.6	0.278
**Health factors**				
** Body mass index**				
Underweight	30.5	30.7	0.1	0.317
Normal	22.5	24.4	1.9	0.004
Overweight	17.5	22.5	5.0	0.271
Obese	16.7	19.8	3.1	0.000
** Self-rated health**				
Good	15.5	15.8	0.3	0.213
Fair	19.8	20.5	0.7	0.041
Poor	33.4	33.3	-0.1	0.162
** Difficulty in ADL**				
No	18.4	18.7	0.3	0.001
Yes	30.8	31.4	0.6	0.660
** Difficulty in IADL**				
No	17.9	16.7	-1.2	0.107
Yes	22.6	24.0	1.4	0.001
** Morbidity status**				
0	19.2	20.7	1.6	0.016
1	21.3	22.1	0.8	0.087
2 +	24.5	24.3	-0.3	0.003
**Household factors**				
** MPCE quintile**				
Poorest	24.2	25.3	1.1	0.109
Poorer	21.8	19.9	-1.9	0.647
Middle	19.9	21.3	1.4	0.316
Richer	19.6	21.3	1.7	0.003
Richest	19.0	22.4	3.4	0.000
** Religion**				
Hindu	21.1	22.2	1.1	0.000
Muslim	23.5	22.8	-0.7	0.801
Christian	15.6	21.5	5.9	0.598
Others	15.0	16.9	1.9	0.505
** Caste**				
Scheduled Caste	22.0	24.7	2.7	0.001
Scheduled Tribe	20.0	17.6	-2.4	0.166
Other Backward Class	22.8	22.1	-0.6	0.046
Others	17.1	20.6	3.5	0.000
** Place of residence**				
Rural	21.9	23.7	1.8	0.000
Urban	18.5	18.2	-0.3	0.008
** Region**				
North	16.3	20.4	4.1	0.035
Central	22.7	25.2	2.4	0.003
East	23.1	25.0	1.9	0.076
Northeast	13.8	15.9	2.1	0.628
West	16.2	16.6	0.4	0.016
South	24.0	22.3	-1.7	0.008

*ADL* Activities of Daily Living; *IADL* Instrumental Activities of Daily Living; *MPCE* Per Capita Monthly Consumption Expenditure

Table 3 shows the results of a logistic regression analysis examining the association of various background characteristics, (individual factors, behavioural factors, health factors, and household factors) and the frailty amongst older adults in India in 2017–18. For each variable, the odds ratio (OR) is reported along with the 95 percent confidence interval (CI) and the p-value. Older adults with a sleep disorder had 1.66 times higher odds of suffering from frailty compared to those without a sleep disorder (*p* < 0.001). As compared to those who frequently engaged in physical activities, those who rarely or never engage in physical activities were highly likely to suffer from frailty. The older adults who reported fair or poor health had higher odds of frailty compared to those who reported good health (Or = 0.73; *P* value = 0.000) The older adults from the eastern region had higher odds of frailty compared to those from the North region (OR = 1.63; *p* value = 0.000).

**Table 3:** Logistic regression estimates for older adults suffering from frailty in India, 2017–18

Background Characteristics	Odds Ratio	CI (95 percent)	*P*-value
		**Lower and Upper values**	
**Sleep disorder**			
No **®**	1		
Yes	1.66	1.47—1.87	0.000
**Individual factors**			
** Age**			
Young-old **®**	1		
Old-old	1.32	1.16 -1.50	0.000
Oldest-old	1.31	1.07 -1.60	0.010
** Education**			
Upto primary **®**	1		
Upto Secondary	0.92	0.81—1.04	0.186
Graduation & above	0.83	0.67—1.04	0.113
** Living arrangements**			
Alone **®**	1		
With spouse	0.77	0.57 -1.04	0.092
With children	0.86	0.66—1.11	0.247
** Marital status**			
Currently married **®**	1		
Widowed	0.89	0.74 -1.06	0.196
Others	0.65	0.43—0.98	0.041
** Working status**			
Yes **®**	1		
No	1.09	0.95—1.24	0.225
** Social engagement**			
1 **®**	1		
2	0.95	0.83—1.09	0.480
3	1.03	0.87—1.21	0.760
**Behavioural factors**			
** Tobacco consumption**			
No **®**	1		
Yes	0.99	0.87—1.12	0.834
** Alcohol consumption**			
No **®**	1		
Yes	1.13	0.98 -1.31	0.088
** Physical activity**			
Frequently **®**	1		
Rare/Never	26.58	19.62—36.02	0.000
**Health factors**			
** Body mass index**			
Underweight **®**	1		
Normal	0.76	0.65—0.89	0.001
Overweight	0.58	0.47 -0.71	0.000
Obese	0.70	0.58—0.84	0.000
** Self-rated health**			
Good **®**	1		
Fair	1.33	1.16 -1.53	0.000
Poor	1.73	1.47—2.04	0.000
** Difficulty in ADL**			
No **®**	1		
Yes	1.37	1.18 -1.59	0.000
** Difficulty in IADL**			
No **®**	1		
Yes	1.14	0.99 -1.30	0.065
** Morbidity status**			
0 **®**	1		
1	1.07	0.93 -1.24	0.325
2 +	1.11	0.95 -1.29	0.200
**Household factors**			
** MPCE quintile**			
Poorest **®**	1		
Poorer	0.97	0.80 -1.17	0.733
Middle	0.98	0.81 -1.19	0.860
Richer	1.1	0.91 -1.33	0.315
Richest	1.15	0.95 -1.40	0.150
** Religion**			
Hindu **®**	1		
Muslim	0.86	0.70 -1.06	0.163
Christian	0.93	0.73 -1.18	0.546
Others	0.74	0.53 -1.02	0.065
** Caste**			
Scheduled Caste **®**	1		
Scheduled Tribe	0.93	0.72 -1.21	0.608
Other Backward Class	0.97	0.81 -1.17	0.779
Others	0.77	0.63—0.93	0.008
** Place of residence**			
Rural **®**	1		
Urban	1.03	0.91 -1.17	0.658
** Region**			
North **®**	1		
Central	1.19	0.94 -1.49	0.143
East	1.63	1.33 -1.99	0.000
Northeast	0.77	0.59 -1.00	0.052
West	0.82	0.66 -1.01	0.063
South	1.09	0.89 -1.32	0.415
**Constant**	0.01	0.01—0.02	0.000

Significant if, *p* < 0.05; UOR: Ref: Reference, CI: Confidence interval

*ADL* Activities of Daily Living, *IADL* Instrumental Activities of Daily Living, *MPCE* Per Capita Monthly Consumption Expenditure

## Discussion

To our knowledge, this is the one of the most detailed effort to systematically and evaluate the relationship between sleep disorder and frailty using a nationally-representative survey data in India. Frailty varies across studies according to the adopted definitions to measure and the tested population. Sleep disorders negatively affect hormonal and metabolic functions by shortening the deep sleep phase, which reduces growth hormone secretion and promotes cortisol secretion [8, 31]. Sleep disorder and frailty has a bi-directional relationship, sleep disorders hamper the growth process and thus lead to muscle loss and eventual frailty, frailty may also cause disturbed sleep due to enhanced inflammations and other secretions in the body [23, 32, 33].

The current analysis showed various covariates predictive of higher prevalence of frailty. It was found, a larger percentage of older women had sleep disorders as compared to older men. These findings align with previous assessments having similar observations [13, 21]. This could be due to poor social support women receive, familial burden, work and responsibilities.[8–10, 34]. This could also be because women tend to have higher C-reactive protein (CRP) and interleukin-6 levels, which act as markers in the etiology of frailty [35] A large number of older adults who had sleeping disorders were also found to be frail. Consonant with previous studies, our results show a pronounced likelihood of older adults with sleep disorders being frail [9, 14, 31, 36].

Studies like [4, 37] reported a higher prevalence of physical frailty among older adults with poor self-reported health, and poor sleeping habits. Studies across the world have shown that older adults having low educational level had higher odds of being frail than those having high educational level [38–41]. The current study’s results also underscore these findings that education plays a vital role in protecting against frailty. Further, older adults not actively participating in any physical activity had significantly higher odds of being frail than older adults who participated in physical activity. This was in line with previous findings [42, 43]. Self-rated health was associated with an increased risk of frailty in older age, [44] SRH in older adults should be recognised as a predictive tool for future frailty. Evidence widely suggests that increased physical activity improves many aspects of sleep and directly combats several frailty symptoms (e.g. slowness, weakness, and low physical activity) [10, 32, 45–48]. There should be studies considering the synergistic effects of sleep and exercise interventions to prevent and reverse frailty.

Though the study is one of the rare attempts at unveiling the association between two of the most related concepts of old age but is not short of limitations, the first being the cross-sectional nature of the study. Hence, strict cause and effect relationship could not be established. Another round of the study would help the researcher establish a better relationship and form a clearer picture. The individuals having a sleep disorder did not undergo any laboratory testing; hence, sleep–wake disturbance could not be objectively confirmed and it could not be truly determined if the underlying cause of frailty was primarily sleeping disorder. Additionally, current study was retrospective. Hence, there is a high chance of recall bias arising. Despite having several limitations, there are various strengths of the study. First, an attempt to study the relationship from a survey of large scale national probability sample of adults (60 years and above). LASI is a large scale comprehensive data set that increases the chance of external validity and generalizability of the current findings. These findings have important public health implications with a longer lifespan and the high prevalence of sleep disorders and frailty in older populations. The results may help the program managers to design relevant strategies to prevent frailty in elderly.

## Conclusions

Sleep is associated with older people’s overall health, and the present study confirms a positive relationship between sleep problem and frailty status. Older women and men both are highly predisposed to faulty sleep and frailty. Since sleep quality is potentially remediable, future frailty prevention interventions should consider sleep complaints. Physical activity, educational attainments affect the frailty status of older adults. The study has clinical relevance since sleep complaints offer a means for identifying those who are vulnerable to frailty and through appropriate intervention, understanding the causes of sleep disorder can help to delay and in some cases reverse frailty.

## Supplementary information

**Additional file 1. **Description of Independent Variables used in the study.

## Data Availability

Data for this study were extracted from the first wave of the Longitudinal Aging Study in India (2017–19) that is freely available in public domain on request through https://www.iipsindia.ac.in/sites/default/files/LASI_DataRequestForm_0.pdf.

## Abbreviations

ADL: Activities of Daily Living
CES-D: Center for Epidemiologic Studies Depression
CRP: C-reactive protein
IADL: Instrumental Activities of Daily Living
LASI: Longitudinal Ageing Study in India
MPCE: Monthly Per-Capita Consumption Expenditure
OBC: Other Backward Castes
OR: Odds Ratio
SC: Schedule Caste
SRH: Self-rated Health
ST: Scheduled Tribe
VIF: Variance inflation factor
