# Supplementary material for: Is there a ubiquitous association between sleep disorder and frailty? findings from LASI (2017–18)
Source: BMC Geriatr. 2023 Jul 12;23:429. doi: 10.1186/s12877-023-04148-x (PMC10339638; doi:10.1186/s12877-023-04148-x)
Supplement: Supplementary file 1 — Additional file 1. Description of Independent Variables used in the study. [file 12877_2023_4148_MOESM1_ESM.docx]

**Supplementary Table-1. Description of Independent Variables used in the study**

| **Socio-demographic characteristics** | **Age-group** | Young old (60-69 years), Old-old (70-79 years), Oldest old (80+ years) |
| --- | --- | --- |
|  | **Sex** | Male, Female |
|  | **Educational status** | No education /Primary not completed, Primary, Secondary, Higher |
|  | **Living arrangement** | Living alone, Living with a spouse, Living with children, Living with others |
|  | **Marital status** | Currently married, Widowed, Others (separated/ divorced/ never married) |
|  | **Working status** | Never worked, Currently working, Currently not working, Retired |
|  | **Social engagement** | Yes, No  Respondents were said to be socially engaged if were actively doing the following. Eat out of the house (Restaurant/ Hotel); Go to park/beach for relaxing/entertainment; Play cards or indoor games; Play outdoor games/ sports/ exercise/ jogging/ yoga; Visit relatives/ friends; Attend cultural performances/ shows/ Cinema; Attend religious functions /events such as bhajan/Satsang/ prayer; Attend political/ community/ organization group meetings; Read books/newspapers/ magazines; Watch television/ listen radio and use a computer for e-mail/net surfing etc. If the older adults was involved in either of the above activities, then the respondent was taken to be socially engaged.  If the older adults was involved in either of the above activities, then the respondent was taken to be socially engaged. |
| **Behavioral characteristics** | Physical activity | Frequent (every day and more than once a week), Rare (once a week, one to three times a month), Never [24,47,48] |
|  | Tobacco Consumption | Yes, No |
|  | Alcohol consumption | Yes, No |
| **Health characteristics** | Body mass index (BMI) | Underweight (less than 18.5), Normal (18.5 to 24.9), overweight (25-29.9), Obese (30 and above) [49] |
|  | Self-rated health (SRH) | Good (Excellent, Very good, and Good), Poor (Fair and Poor) |
|  | Difficulty in ADL | Yes (refers to difficulty in normal daily self-care activities, such as movement in bed, changing position from sitting to standing, feeding, bathing, dressing, grooming, personal hygiene, etc.), No (refers to no difficulty in above activities) [24] |
|  | Difficulty in IADL | Yes (Instrumental activities of daily living are not necessarily related to the basic functioning of a person, but they let an individual live independently in a community. These tasks are necessary for independent functioning in the community. If the respondent reported any difficulty in the above IADL’s then difficulty in IADL was coded as yes otherwise, No [24] |
|  | Morbidity status | 0 “No morbidity,” 1 “Anyone morbid condition,” and 2+ “Co-morbidity” |
| **Household characteristics** | MPCE | Poorest, Poorer, Middle, Richer, Richest  MPCE) quintile was assessed using household consumption data. Sets of 11 and 29 questions on the expenditures on food and non-food items, respectively, were used to canvas the sample households. Food and non-food expenditures have been standardized to the 30-day reference period. It is used as the summary measure of the consumption [24] |
|  | Region | North, Central, East, Northeast, West, and South |
|  | Religion | Hindu, Muslim, Christian, Others |
|  | Caste | ST, SC, OBC, Others |
|  | Place of residence | Rural, Urban |
